# Antioxidant and Anti-Inflammatory Properties of Phytochemicals Found in the *Yucca* Genus

**DOI:** 10.3390/antiox12030574

**Published:** 2023-02-24

**Authors:** Erick Bahena Culhuac, Aristide Maggiolino, Mona M. M. Y. Elghandour, Pasquale De Palo, Abdelfattah Z. M. Salem

**Affiliations:** 1 Facultad de Ciencias, Universidad Autónoma del Estado de México, Toluca 50000, Estado de México, Mexico; 2 Department of Veterinary Medicine, University of Bari A. Moro, 70010 Valenzano, Italy; 3 Facultad de Medicina Veterinaria y Zootecnia, Universidad Autónoma del Estado de México, Toluca 50000, Estado de México, Mexico

**Keywords:** *Yucca* genus, ethnobotany, phytochemicals, anti-inflammatory, antioxidant

## Abstract

The *Yucca* genus encompasses about 50 species native to North America. Species within the *Yucca* genus have been used in traditional medicine to treat pathologies related to inflammation. Despite its historical use and the popular notion of its antioxidant and anti-inflammatory properties, there is a limited amount of research on this genus. To better understand these properties, this work aimed to analyze phytochemical composition through documentary research. This will provide a better understanding of the molecules and the mechanisms of action that confer such antioxidant and anti-inflammatory properties. About 92 phytochemicals present within the genus have reported antioxidant or anti-inflammatory effects. It has been suggested that the antioxidant and anti-inflammatory properties are mainly generated through its free radical scavenging activity, the inhibition of arachidonic acid metabolism, the decrease in TNF-α (Tumor necrosis factor-α), IL-6 (Interleukin-6), iNOS (Inducible nitric oxide synthase), and IL-1β (Interleukin 1β) concentration, the increase of GPx (Glutathione peroxidase), CAT (Catalase), and SOD (Superoxide dismutase) concentration, and the inhibition of the MAPK (Mitogen-Activated Protein Kinase), and NF-κB (Nuclear factor kappa B), and the activation of the Nrf2 (Nuclear factor erythroid 2–related factor) signaling pathway. These studies provide evidence of its use in traditional medicine against pathologies related to inflammation. However, more models and studies are needed to properly understand the activity of most plants within the genus, its potency, and the feasibility of its use to help manage or treat chronic inflammation.

## 1. Introduction

The *Yucca* genus belongs to the Agavoideae subfamily, a subfamily that is commonly used in traditional medicine thanks to its anti-inflammatory, antimicrobial, and antiparasitic properties [1]. It encompasses about 40 to 50 species, most of which are native to southern North America. These plants have been used for centuries to treat different ailments [2]. These benefits led to the approval by the FDA (Food and Drug Administration) of the use of *Yucca* species in various products, especially in dietary supplements [3]. These benefits have attracted research into the genus, which has demonstrated the presence of many biological properties [2].

One of *Yucca*’s most notable properties is its anti-inflammatory activity. Inflammation is a physiological procedure generated by the immune system in response to tissue injury, stress, pathogens, or toxic compounds [4]. However, in some cases, inflammation can become harmful to the body, such as chronic inflammatory diseases [5], and in those cases, the inflammatory response must be suppressed. The inflammatory process generates reactive oxygen species (ROS) and reactive nitrogen species (RNS), which can cause oxidative stress [5]. Oxidative stress occurs when those oxidative molecules surpass the antioxidant system, and this will damage or affect the function of proteins, lipids, DNA, or RNA [6]; for the same reason, treatment with antioxidants has been shown to help treat inflammatory diseases, such as inflammatory bowel disease [7].

Treatment against these diseases is usually anti-inflammatory. Unfortunately, there are some drug-induced side effects that make some treatments inadequate [7], and there has been a tendency to use traditional plant-based remedies to partially treat inflammatory diseases. Some of these plants have been *Yucca* species, as they are popularly used to treat arthritis since they counteract some effects of this disease. All of the biological properties of plants are due to the high concentration of phytochemicals [2]. However, to better understand the anti-inflammatory and antioxidant activities within the genus, it is necessary to understand the phytochemical composition and how they act.

Therefore, the objective of this work is to research the phytochemical composition of the species and the anti-inflammatory and antioxidant properties of these molecules. The literature was explored during the period from 2000 to 2022, although the oldest papers were used for the historical context. In this way, it will be possible to know the possible molecules responsible for these effects and their mechanisms of action.

## 2. *Yucca* Genus

*Yucca* species are native from North and Central America [8], and these plants are tolerant to drought, wind, and salt, which is why most of these species thrive in the arid zones of the USA and Mexico [9]. The genus is known for its obligate pollination mutualism with *Yucca* moths, where the moths provide *Yucca* with pollen while using the flower to oviposit [8]. Hybridization is common among species, which makes species classification difficult [10].

The literature indicates that the *Yucca* genus is composed of about 50 species. In particular, “The Plant List” establishes 49 species with an “accepted” status [11]. Although each species has each morphological characteristic, in general, they are long-lived perennials, tree-shaped, with white flowers, and sword-shaped leaves that grow in rosettes [8].

## 3. Ethnobotanical Use

Since ancient times, *Yucca* species have been used by natives for many purposes. Some species, such as *Yucca schidigera* Roezl ex Ortgies, were used for bowstrings, nets, ropes, mats, sandals, and clothing [12]. It is also used as food; an example is that in eastern Costa Rica, there is a tradition where they eat the Itabo flower (*Yucca elephantipes* Regel) at Easter [13]. Most importantly, these species have been used in traditional medicine. The Cheyenne cultures used *Yucca glauca* Nutt to stimulate hair growth, and in skin conditions [14]. New Mexico healers use it to treat asthma and headaches [15]. There are also more modern claims of its ethnobotanical use to treat asthma, rheumatism, gonorrhea, sunburns, arthritis, etc. [2,16].

These ethnobotanical uses have led to the emergence of research on their biological activities. Starting in 1975, it was tested as a treatment to manage arthritis [17]. Ever since, hypocholesterolemic activity [18], antimicrobial activity [19], antiprotozoal activity [20], antioxidant activity [21], anti-inflammatory activity [16], and many others have been tested for this genus. The literature especially highlights its anti-inflammatory and antioxidant activity. As mentioned before, all these properties are generated through phytochemicals. Therefore, knowing these molecules allows us to gain a better understanding of their anti-inflammatory and antioxidant activity.

## 4. Antioxidant and Anti-Inflammatory Activities

**Figure 1:**
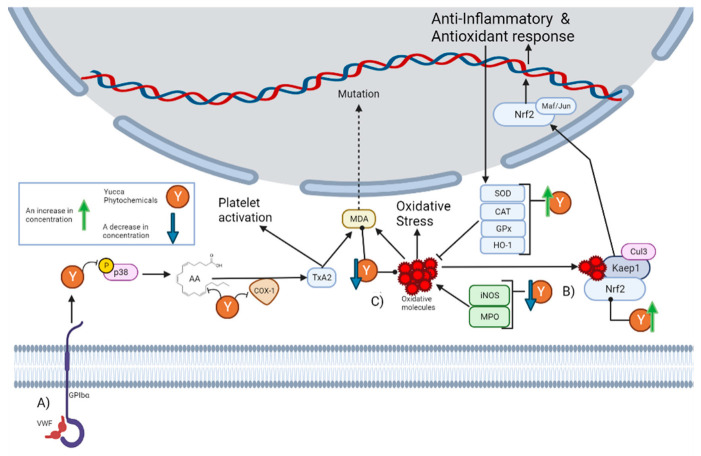
This figure illustrates how the *Yucca* genus phytochemicals influence anti-inflammatory and antioxidant processes through: (**A**) The platelet activation pathway begins with VWF binding to the GPIbα, which generates the phosphorylation of p38. This leads to the subsequent release of AA, which is converted by COX-1 to the platelet activator TxA2, forming MDA as a residue. MDA can react with DNA-inducing mutations. *Yucca* genus phytochemicals can interrupt this pathway by preventing the generation of arachidonic acid, decreasing the concentration of p38, or inhibiting its phosphorylation and COX-1 activity. (**B**) *Yucca* genus phytochemicals have the capacity to eliminate oxidative stress. This is due to the upregulation of HO-1, GPx, CAT, and SOD. By reducing the concentration of MPO and iNOS. The free radical scavenging activity of many *Yucca* genus phytochemicals reduces the concentration of oxidative molecules, such as MDA. (**C**) Nrf2 is regulated by Keap1 and the Cul3 ubiquitin E3 ligase complex. Nrf2 can be activated by oxidative molecules that modify the cysteine residues of Keap1. Once Nrf2 is free, it translocates to the nucleus, and heterodimerizes with small Maf or Jun proteins to generate an antioxidant and anti-inflammatory response. *Yucca* genus phytochemicals can activate this pathway. The figure was created with BioRender.com.

Despite historical ethnobotanical use, its continuous mention in the literature, and the popular notion of its anti-inflammatory properties, a minimal number of current studies have explored these properties. The most mentioned activity in the literature is the anti-platelet effect of *Yucca schidigera* Roezl ex Ortgies. Platelets are specialized blood cells that play an important role in inflammation. Platelets are capable of upregulating leukocyte functions and releasing proinflammatory cytokines [22]. It has been reported that alcoholic extract from *Yucca schidigera* Roezl ex Ortgies significantly decreases the various steps of thrombin-platelet activation [23]. The platelet activation pathway begins with stimulation originating from the von Willebrand factor (VWF) to the platelet adhesion receptor glycoprotein (GPIbα), which generates the phosphorylation of P38. This leads to the subsequent release of arachidonic acid (AA), which is then converted into the platelet activator thromboxane A2 (TxA2) by cyclo-oxygenase 1 (COX-1) [24]. As can be seen in Section 5, there are phytochemicals within the genus that can interrupt this pathway. First, n-3 fatty acids can prevent the generation of arachidonic acids [25]. Then, there are molecules that can decrease the concentration of p38, while others inhibit its phosphorylation. There are also molecules that inhibit COX-1 activity and others that decrease its concentration. In fact, it has been reported that the alcoholic extract from *Yucca schidigera* Roezl ex Ortgies inhibits COX-1 and COX-2 (Cyclooxygenase-2) in vitro [26]. This is graphically represented in Figure 1A.

The anti-inflammatory effect of *Yucca gloriosa* L., specifically its inhibitory potential against Ovalbumin-Induced Airway Hyperresponsiveness in mice [16] has also been reported. Thus, the alcoholic extract of *Yucca gloriosa* L. was administered orally at doses of 50, 100, or 200 mg/kg for 7 days 1 h before each sensitization with ovalbumin. Pretreatment with *Yucca gloriosa* L. significantly decreased the concentrations of TNF-α, IL-6, interleukin-13 (IL-13), and leucocyte count.A similar effect to that reported for various phytochemicals found within the genus, as can be seen in Section 5. Repeated exposure to ovalbumin mimics the symptoms of asthma [27]. Thus, it suggests a possible use of *Yucca gloriosa* L. against asthma.

**Figure 2:**
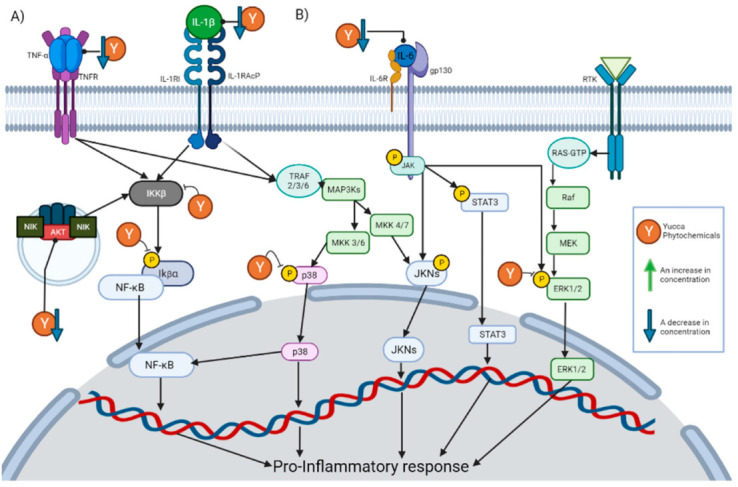
This figure illustrates how the *Yucca* genus phytochemicals influence anti-inflammatory and antioxidant processes through: (**A**) The NF-kB pathway can be canonically activated by cytokines, such as TNF-α and IL-1 β, or uncanonically by the endosome complex containing NIK, AKT, and MAC. The pathway begins in the activation of IKK β, which phosphorylate IκBα. This results in the release of NF-kB dimers, translocating to the nucleus and activating the transcription of proinflammatory proteins. *Yucca* genus phytochemicals can interrupt this pathway by reducing the concentration of TNF-α, IL-1, and AKT, or the inhibition of IKK activation and phosphorylation IκBα. (**B**) There are 3 MAPK pathways, the ERK1/2, JNK, and p38 MAP kinase. The p38 MAPK pathway begins the activation of receptors, such as TNFR or the IL1R superfamily. This activation will generate the phosphorylation of TRAF 2/3/6 which in turn activates MAP3Ks, which phosphorylates MKK3 or MKK6, and those molecules will activate p38. P38 positively regulates a pro-inflammatory response. In addition, p38 partially modulates the activation of the basal transcription factors that interact with NF-κB. *Yucca* genus phytochemicals can reduce p38 phosphorylation, inhibiting the signaling pathway. The activation of JNK begins with the phosphorylation of MAP3Ks, which subsequently phosphorylates MKK7 or MKK4, and then phosphorylates the JNK kinases. JNK positively regulates a pro-inflammatory response. IL-6 can activate JNK pathway and STAT3 pathways by activating membrane-bound gp130, which will cause JAK enzymes to phosphorylate proteins such as STAT3. *Yucca* genus phytochemicals decrease the activation of this pathway. ERK1/2 signaling begins with the binding of a ligand to the RTK; this will activate the G-protein known as Ras. Ras directly binds to Raf and activates it, then Raf activates MEK, and MEK phosphorylates ERK1/2 so it can enter the nucleus and activate transcription factors. *Yucca* genus phytochemicals have the capacity to suppress this signaling pathway. The figure was created with BioRender.com.

Specifically, the decrease in the TNF-α concentration is the effect with the highest incidence within these compounds. This is expected due to the key role that it plays in inflammation. TNF-α is released by a wide variety of immune cells so that it can bind to its receptors and activate different pathways. Once TNF-α has bound to its receptor, it promotes the formation of a complex capable of activating IκBα kinase (IKK) [28]. Once activated, IKK will phosphorylate IκB (Inhibitor of nuclear factor kappa B), which would cause the degradation of IκB and the release of NF-κB [29]. The release of NF-κB will allow its nuclear translocation and the activation of said signaling cascade. There are other pathways that can be induced by TNF-α, such as the c-Jun amino terminal kinase (JNK), p38-MAPK, extracellular signal-regulated kinase 1/2 (ERK1/2) and AKT pathways [28]. Inhibition of all these pathways has also been reported for the phytochemicals described in Section 5. This is graphically represented in Figure 2A.

Another of the effects with a high incidence is a decrease in the concentration of IL-6. IL-6 exerts its activity by binding to the IL-6 receptor, thereby activating membrane-bound gp130. This causes JAK enzymes to phosphorylate gp130, thus generating docking sites for proteins, such as STAT3, to bind to and be phosphorylated by JAK enzymes [30]. In this way, signaling pathways, such as MAPK and JAK/STAT3, are initiated. This is graphically represented in Figure 2B.

In addition to these proinflammatory mediators, treatment with *Yucca gloriosa* L. also decreased the concentration of the oxidative markers nitric oxide (NO), myeloperoxidase (MPO), and malonaldehyde (MDA) [16]. As can be seen in Section 5, the decrease in NO concentration is frequently mentioned, as in Yuccaol A [28]. NO acts as a mediator of inflammatory processes. It is synthesized by the enzymes nitric oxide synthase (NOS) from L-arginine, especially in the context of inflammation; the inducible isoform of NOS (iNOS) is mainly responsible for its production [31]. Although there are several pathways that result in the expression of iNOS, the literature highlights that the transcription factors NF-kB and STAT-1α are essential for its expression in most cases [32]. The main role of NO in inflammation is to react with superoxide anions to form peroxynitrite [31]. However, it is also involved in some regulatory mechanisms since it can react with transition metals or induce nitrosylation within proteins and regulate their activity [31]. Excessive production of NO is present in pathologies, such as hypertension or atherosclerosis [33]. The ability to inhibit iNOS expression stands out due to Yuccaol C, a molecule proper to the genus that has the capacity to reduce the expression of iNOS in J774.A1 macrophages at 1 µM [34]. There are also other phytochemicals within the genus that produce the same effect (Figure 1B).

In addition to NO reduction, many phytochemicals also reported a significant capacity for reducing the concentration of MDA and MPO, known biomarkers of oxidative stress. MPO is a key enzyme in the elimination of pathogens within phagolysosomes of neutrophils, since it generates powerful oxidizing species, such as hypochlorous acid (HOCl), from the catalysis of the reaction of chloride with hydrogen peroxide [35]. HOCl can generate modifications against pathogens’ lipids, DNA, and proteins, but due to this activity, it can also damage host tissue and is involved in inflammatory diseases, such as atherosclerosis [36]. HOCL can react with phosphatidylethanolamines (PEs) and form PE-monochloramine or PE-dichloramine, which are plausible initiators of lipid peroxidation [37]. Lipid peroxidation is the process by which oxidants attack lipids, especially polyunsaturated fatty acids (PUFAs), in the lipid membrane [38]. The peroxidation of PUFAs forms lipid peroxides, which are unstable and decompose to a series of compounds, such as MDA. MDA is formed by the decomposition of arachidonic acid AA during the biosynthesis of TxA2 or by bicyclic endoperoxides during polyunsaturated fatty acid peroxidation [38]. MDA levels are a widely used indicator of lipid peroxidation and oxidative stress, and high levels are associated with various health disorders, such as in lung cancer patients or glaucoma patients [39]. This may be due to the fact that MDA reacts with DNA to form adducts, which have been reported to frequently induce mutations in oncogenes [40]; this is graphically represented in Figure 1A.

There are other reports of the antioxidant activity of the *Yucca* genus against oxidative stress. A commercially available food additive known as Sarsaponin 30^®^ has been reported to have a protective effect against nitrite-induced oxidative stress in rats [41]. Rats were pretreated with Sarsaponin 30^®^ for 4 weeks prior to the nitrite intoxication in doses of 100 ppm. Said pretreatment reduced the concentrations of MDA and NO in the tissue and in glutathione (Figure 1B).

In addition to rats, dietary supplementation with *Yucca schidigera* Roezl ex Ortgies *a* has also shown antioxidant capacity against oxidative stress in fish. In Oreochromis niloticus Biodust^®^; other food additives from *Yucca schidigera* Roezl ex Ortgies alleviate growth arrest, intestinal dysfunction, and oxidative damage induced by heat stress [42]. This is done by the downregulation of the ubiquitin-proteasome system, TNF-α, IL-1β, and interleukin 8 (IL-8), as well as by enhancing the Nrf2 signaling pathway. As can be seen in Section 5, the decrease of IL-1β concentration is an effect well represented through phytochemicals within the genus. IL-1 refers to two separate cytokine genes, IL-1α and IL-1β, that bind to the same receptors and stimulate similar proinflammatory signals [43]. For IL-1β to be excreted, its precursor must be processed by caspase-1 from the NALP3 inflammasome and excreted by the ATP/P2X7R influx [44]. In the same way, within the described phytochemicals of the genus, there are reports of the ability to inhibit NALP3 inflammasome formation, which would prevent the excretion of IL-1β. Once excreted, it will exert its activity by binding to the extracellular IL-1 type I receptor (IL-1RI), which will lead to the recruitment of IL-1R accessory protein (IL-1RAcP) and other adapters, and thus activate the NFκB, JNK, ERK, or MPAK signaling pathways [43], and this graphical represented in Figure 2A.

Another case with fish was with *Cyprinus carpio*, where they were fed an extract of *Yucca schidigera* Roezl ex Ortgies at doses of 200 or 400 mg/kg for 8 weeks, which improved their growth and intestinal antioxidant status [45]. This is due to an increase in the mRNA levels of GPx, CAT, SOD, and Nrf2, in addition to a reduction in the levels of IL-1β and IL-6. GPx, CAT, and SOD are known as front-line antioxidant defense systems. This is because ROS molecules are the most abundant oxidizing molecules within cells, especially molecules such as superoxide anions. Moreover, SOD can transform these superoxide anion molecules into hydrogen peroxide (H_2_O_2_) and O_2_, so that subsequently CAT or GPx catalyzes the reduction of H_2_O_2_ to water, thus eliminating the oxidative danger of these molecules [46]. It should be noted that the increase in these antioxidant enzymes is one of the most reported effects within the phytochemicals reported in Section 5, as many managed to increase the concentration of these 3 enzymes (Figure 1B).

As it can be seen through Section 5 that the reported effect of *Yucca* extracts, the main pathways involved in its anti-inflammatory and antioxidant effect are the inhibition of MAPK, and NF-κB, and the activation of the Nrf2 signaling pathway.

### 4.1. Inhibition of NF-κB Signaling Pathway

The NF-κB is a family of transcription factors that coordinate one of the most common proinflammatory signaling pathways. Within the phytochemicals in Section 5, there is constant mention of the inhibition of this pathway (Figure 2A). The family of NF-κB has 5 members: RelA, c-Rel, RelB, p50, and p52. RelA, RelB, and c-Rel share a transactivation domain that makes them capable of promoting transcriptional activation, while p50 and p52 act as coactivators [47]. There are 2 variations of this pathway, the canonical one where RelA and p50 are responsible for promoting the transcription of target genes, and in the non-canonical RelB and p52 [48].

The canonical NF-kB pathway is primarily a response to proinflammatory cytokines, such as TNF-α and IL-1, and it has an important role in chronic inflammatory diseases [49]. The pathway begins with the activation of receptors, such as TNFR and IL-1RI, which will generate a series of steps resulting in the activation of IKKβ [48]. The IKKβ will phosphorylation IκBα, which results in the release of the sequestered RelA-p50 dimers. Once these dimers translocate to the nucleus, they activate the transcription of receptors and proinflammatory cytokines involved in the inflammatory response [48]. As mentioned above, the inhibition of IKK activation and phosphorylation IκBα are an abundant effect between phytochemicals described in Section 5.

The non-canonical NF-kB pathway begins with the activation of the TNFR superfamily members, or the formation of an endosome complex containing NIK, AKT, and MAC, to stabilize and accumulate NIK. The NIK (NF-kB inducing kinase) with IKKα will induce the phosphorylation of the precursor of p52, resulting in the formation of RelB/p52 dimer [49].

### 4.2. Inhibition of the MAPK Signaling Pathway

The MAPK superfamily is one of the major mechanisms used in signaling pathways and is characterized by its activation through the dual phosphorylation on adjacent threonine and tyrosine residues [50]. In inflammation, the activation of receptors triggers the MAPK pathways, and transcription factors are phosphorylated and activated, such as NF-κB [51]. There are 3 well-known MAPK pathways, the ERK1/2, JNK, and p38 MAP kinase, all of which activate proinflammatory stimuli [52]. ERK1/2 signaling begins with the binding of a ligand to the receptor tyrosine kinase (RTK); this activates G-protein kwon as Ras. Ras directly binds to Raf and activates it, then Raf activates MEK, and MEK phosphorylates ERK1/2 so it can enter the nucleus and activate transcription factors [53]. As can be seen in Section 5, there are molecules within the *Yucca* genus that have shown the capacity to suppress this signaling pathway.

The p38 MAPK pathway begins the activation of receptors, such as toll-like receptors, TNFR, or the IL1R superfamily, to inflammatory stimuli. This activation generates the phosphorylation of TRAF 2/3/6 (TNF receptor-associated factor), which in turn activates MAP3Ks, such as TAK1. Then, MAP3K phosphorylates MKK3 or MKK6, and those molecules activate p38 [54]. Many of the pro-inflammatory responses, such as TNF-α, IL-1β, IL-6, IL-8, and COX-2, are positively regulated by p38 [51]. This pathway can regulate the NF-κB-dependent gene expression because p38 partially modulates the activation of basal transcription factors that interact with NF-κB [51]. Phytochemicals within the *Yucca* genus can reduce p38 phosphorylation and inhibit the signaling pathway.

There are 3 types of JNK proteins JNK1 (encoded by MAPK8), JNK2 (encoded by MAPK9), and JNK3 (encoded by MAPK10), where JNK1 and JNK2 are found in almost all cells [55]. This signaling pathway, as the other two can be triggered by proinflammatory cytokines. The activation of JNK begins with the phosphorylation of MAP3Ks, which subsequently phosphorylates MKK7 or MKK4, and then phosphorylates the JNK kinases [56]. JNK regulates the activity and maturation of T cells, as well as pro-inflammatory cytokines such as IL-6 and TNF-α, and therefore this pathway is related to chronic inflammatory disorders [57]. Within the genus, there are molecules that decrease the activation of this pathway (Figure 2B).

### 4.3. Activation of the Nrf2 Signaling Pathway

Nrf2 is a transcription factor that regulates the expression of antioxidant and anti-inflammatory proteins, and it is considered a modulator of species longevity [58]. Its anti-inflammatory effect is due to an indirect control of NF-kB activity and a direct control of IL-6 and IL-1β expression [59]. In fact, under normal inflammatory conditions, Nrf2 expression is activated by NF-kB to initiate a slow response that can stop the NF-kB inflammatory response [60]. Nrf2 is considered the major regulator against oxidative stress, as it regulates the expression of antioxidant response element genes, such as SOD, GPx, NADP(H) quinone oxidoreductase (NQO1), and heme oxygenase (HO-1) [61]. It also regulates Phase II of xenobiotic metabolism, where it transforms carcinogenic intermediates, generated by Phase I of xenobiotic metabolism, into less toxic metabolites [62].

Nrf2 is regulated by Kelch-like ECH-associated protein 1 (Keap1) and the Cullin 3 (Cul3) ubiquitin E3 ligase complex. Keap1 sequester Nrf2 and functions as an adaptor, so the Cul3 complex ubiquitinates Nrf2 to facilitate its proteasomal degradation [63]. Nrf2 can be activated by oxidative molecules modifying the cysteine residues of Keap1, stabilizing Nrf2-Keap1 interaction, and preventing Nrf2 ubiquitination [61,64]. Therefore, new Nrf2 could be synthetized without Kaep1 being able to sequester it. Nrf2 binds to Keap1 through a high-affinity ETGE motif, so proteins with this motif can interact with Keap1 and prevent Nrf2 sequestering [64]. Once Nrf2 is free, it translocates to the nucleus, and heterodimerizes with small Maf or Jun proteins to upregulate or inhibit target genes [61]. The activation of this pathway is one of the most reported effects throughout this genus of phytochemicals, by increasing the concentration of Nrf2 or inhibiting Keap1 (Figure 1B).

One gene regulated by Nrf2 is HO-1 (Heme oxygenase 1). The main function of HO-1 is to catalyze Haem (an iron-containing porphyrin) degradation; it uses cytochrome P450 reductase to transform Haem, NADPH, and O2 to biliverdin, carbon monoxide, ferrous iron (Fe^2+^), NADP+, and H_2_O [65]. However, it has also been shown to have anti-inflammatory properties. They have been shown to help chronic inflammation, along with Nrf2, to inhibit the adhesion of inflammatory cells by downregulating the expression of cell adhesion molecules, such as vascular cell adhesion molecule 1 (VCAM1) [60]. This could explain the ability of some reported phytochemicals to decrease the expression of VCAM1.

### 4.4. Free Radical Scavenging Activity

Finally, there are relatively abundant reports in the literature on extracts from the *Yucca* genus with free radical scavenging activity in vitro. *Yucca aloifolia* L. leaf extracts with MeOH, CHCl3, EtOAc, nBuOH, and n-hexane solvent were tested for their radical scavenging activity [66]. Were *Yucca aloifolia* L. MeOH showed the highest potential by having an activity versus control of 74% in the 1,1-diphenyl-2-picrylhydrazyl (DPPH) assay and an inhibition of 64% in the linoleic acid peroxidation assay. *Yucca schidigera* Roezl ex Ortgies radical scavenging activity was tested with TEAC (Trolox Equivalent Antioxidant Capacity) assay and had trolox equivalents (TE) values of 1.78 [67,68] and 5.78 mM [69], respectively. *Yucca baccata* Torr. butanolic extract showed a 29.18 (μg TE/mg) in DPPH assay, 121.8 (μg TE/mg) in TEAC assay, 33.41 (μg TE/mg) in ferric reducing antioxidant power (FRAP) assay, and 156.84 in oxygen radical absorbance capacity (ORAC) assay [70]. These reports are consistent with the radical scavenging activity observed in phytochemicals observed in Section 5 (Figure 1B).

## 5. Phytochemistry

For thousands of years, mankind has used plants to treat various ailments. This knowledge has been passed down through hundreds of generations and remains the main form of health care for more than 4 billion people today [71]. Phytochemicals naturally protect the plant from environmental hazards, pathogenic attacks, or grant characteristics, such as its aroma and flavor. Due to these functions’, plants have the capability to produce a wide range of molecules, where factors such as soil pH, light, temperature, or stress will change its chemical composition [71]. Many of these molecules will have similar proprieties, especially those that are closely related.

Due to that, in recent years, there has been a trend in countries such as China where plants are being used to generate new drugs. Some phytochemicals are able to modulate inflammation and oxidative stress at the same time, since these two physiological phenomena often share the same pathways and intensify each other. An example of this is that ROS can act as an inflammatory signaling molecule, and in turn, inflammation can induce oxidative stress and reduce cellular antioxidant capacity [72].

Out of the documentary research, 365 molecules were found in the literature, of which 92 had antioxidant or anti-inflammatory reported activity. Of these molecules, 51 can be classified as Phenolic Compounds, 13 as Glycosides, 7 as Saponins, 9 as Fatty acids, 5 as Terpenes, 3 as Tocopherol, 2 as Dicarboxylic acid, 1 as Phytosterol, and Xanthones. The antioxidant and anti-inflammatory activities reported in the literature of the phytochemicals found in the *Yucca* genus can be seen through Section 5.1, Section 5.2, Section 5.3, Section 5.4 and Section 5.5

### 5.1. Phenolic Compounds

Phenolic compounds are phytochemicals that are characterized as containing an aromatic ring bonded to some hydroxyl groups in their structure. Plants can produce a wide variety of phenolic compounds [73]. These compounds play an important role in defense mechanisms against pathogens and stress conditions, such as drought, salinity, and UV [74]. This role is due, in part, to the structural capacity to capture free radicals and chelate metals, which protect the plant from oxidizing molecules [73]. These molecules maintain this antioxidant capacity when consumed, but as can be seen in Table 1, this is not the only reason behind their antioxidant or anti-inflammatory properties. Many of these molecules can downregulate inflammatory pathways, such as NF-kB, and upregulate antioxidant pathways, such as Nrf-2. A behavior that has been described similarly to non-steroidal anti-inflammatory drugs, the most commonly used drugs against inflammation [75].

Within the *Yucca* genus, there is great diversity and concentration of phenolic compounds. Specifically, unique phenolic derivatives with potent antioxidant activity have been found in *Yucca gloriosa* L. (gloriosaols) and *Yucca schidigera* Roezl ex Ortgies (yuccaols) [34,69]. Among these unique molecules of the genus, Yuccaol C stands out because it prevents NF-kB activation and inhibits iNOS expression and NO release in a dose-dependent manner [34].

### 5.2. Saponins

Saponins are amphiphilic compounds that have a saccharide chain attached to a steroid or triterpenoid [157]. These compounds are involved in plant development and protection, where they are synthesized in response to pathogens, insects, or herbivores [158]. They are found in legume seeds in the human diet, and various positive effects on health are attributed to them [157]. In fact, since 1950, these molecules have been used to produce steroidal hormones and drugs [159]. As can be seen in Table 2, saponins have the capacity to decrease the levels of proinflammatory cytokines, especially steroidal saponins, due to their similarity to steroid hormones. This similarity allows some saponins to act as agonists to the glucocorticoid receptor, which generates glucocorticoid-like effects [160]. These types of molecules are found in a high content within the *Yucca* genus and are widely used in the food, pharmaceutical, and cosmetic industries [161]. As with the phenolic compounds, in *Yucca schidigera* Roezl ex Ortgies, new saponins have been found: *Yucca spirostanosides* [162].

### 5.3. Glycosides

Glycosides are a large structurally diverse group of phytochemicals; they have 2 units a small metabolite (aglycone) and a sugar (glycone) [176]. When plants add sugar to small metabolites, it improves their biodistribution, metabolism, and storage [177]. Most of its biological activities come from the “small metabolite”, but the addition of sugar will change the magnitude of the activity. An example of this is that rutin (Quercetin 3-rutinoside) has higher anti-inflammatory activity than its aglycone part, quercetin [178]. This difference may be due to its absorption and metabolism, where glycosides are mostly absorbed in the small intestine after deglycosylation, which allows the metabolite to enter the liver and then be excreted to the blood [179]. As can be seen in Table 3, its antioxidant and anti-inflammatory activity is well known.

### 5.4. Fatty Acids

Fatty acids are lipid structures composed of a long carbon chain with a carboxyl group at one end and a methyl group at the other end [201]. If this structure has a double bond, it is classified as “Unsaturated fatty acids”. Plants mainly produce unsaturated fatty acids. These can be synthesized by plants as part of the various defense systems against biotic and abiotic stresses [202]. Fatty acids also function as modulators of cell membranes, as energy reserves, as extracellular barriers, and as precursors of signaling molecules [203]. As can be seen in Table 4, their anti-inflammatory and antioxidant properties are well known. This effect depends on the position of the first double bond within the carbon chain. If it occurs in the sixth (n-6), it will be considered pro-inflammatory because it is a precursor of arachidonic acid [25]. If it occurs in the third (n-3), it will be considered anti-inflammatory because it will compete as a substrate for n-6 metabolism [25]. On the other hand, these structures are susceptible to oxidize, and for the same reason, they work as antioxidants. Within the *Yucca* genus, it has been reported that *Yucca aloifolia variegate* L. contains more saturated fatty acids than unsaturated, constituted mainly by palmitic acid and palmitoleic acid [87].

### 5.5. Other Phytochemicals

Among the phytochemicals that were found at a lower frequency, the terpenes stand out with the anti-inflammatory effect. Terpenes are the most abundant and diverse class of phytochemicals; structurally, they are made up of isoprene molecules (Table 5). They have a wide range of functions, from primarily being part of plant structures to being quinones in electron transfer [215]. On the other hand, tocopherols stand out for their antioxidant activity. These molecules are exclusively synthesized in photosynthetic organisms and consist of a chromanol head group with one, two, or three methyl groups, and an isoprenoid [216]. α-Tocopherol is the major vitamin E component and one of the most important antioxidant regulatory mechanisms [217].

### 5.6. Availability of Reported Phytochemicals

It is worth noting that the presence by itself of phytochemicals does not guarantee that it will generate the desired biological effect. As with drugs, the quantity of the phytochemical dictates its efficacy. There are many factors that could alter the quantity of phytochemicals. Phytochemicals are mostly generated in response to external stimuli [71]. Thus, all external stimuli alter the synthesis of phytochemicals. In the same way, there will be differences depending on the tissue. In addition, plant tissue may undergo postharvest changes [244]. Then, the extraction of phytochemicals will alter the availability. Here, factors such as the solvent, temperature, time, and pH, among others, will influence the type and amount of phytochemicals obtained [245]. In general, there are a small number of reports assessing the quantity of *Yucca* phytochemicals in the literature. The same is true regarding the difference between extraction and improvement in phytochemical concentration. Within the reports included here, the great variability caused by the factors previously described is notorious. This can be seen in Table 6. Specifically, the difference can be seen when comparing the quantity of resveratrol, 3,3’,5,5’-tetrahydroxy-4-methoxystilbene, Yuccaol A, and Yuccaol C obtained between both extraction methods. Despite the differences in concentrations and types of phytochemicals, the presence of multiple phytochemicals with similar biological effects would suggest a robustness that would allow for the prevalence of their antioxidant and anti-inflammatory activity.

Finally, another important factor related to the availability of phytochemicals is microbiota. The gut microbiota metabolizes most molecules consumed, including drugs or phytochemicals. In the intestine, phytochemicals are degraded by microbes and absorbed by tissues [246]. Some phytochemicals need to be metabolized by the gut microbiota in order to generate its biological effect [247]. Poorly absorptive phytochemicals can undergo structural modifications that improve their bioavailability [246,247]. This especially applies to glycosides, as mentioned above. Glycosides have low bioavailability and bioactivity until their aglycone is deglycosylated by gut microbiota [246,247]. This modification through gut microbiota has been reported to have a role in some antioxidant and anti-inflammatory effects. This is especially true through Nrf2, as the genus *Lactobacillus* capable of stimulating its activation through small molecules [248]. One example of this is the biotransformation of caffeic acid, a phytochemical that can be found in the *Yucca*, into 4-vinyl-catechol. This is an activator of Nrf2 [249]. It has also been reported that treatment with pre-fermented Angelica sinensis activates Nrf2 signaling better than treatment with non-fermented Angelica sinensis in mice [250]. It also increases the level of bacteria related to Nrf2 signaling, such as *Lactobacillus*. Thus, the fermentation of phytochemicals through bacteria, such as Lactobacillus, is key for the efficient activation of Nfr2. From the abundance of reports on *Yucca* phytochemicals activating Nrf2, it could be assumed that other phytochemicals follow the pathway of phytochemicals and are metabolized by bacteria into Nrf2 activators.

## 6. Future Perspectives

Although there is research on the anti-inflammatory and antioxidant properties of the *Yucca* genus, there are still many unknowns to be resolved. First, most of the research related to the anti-inflammatory and antioxidant properties has only focused on *Yucca schidigera* Roezl ex Ortgies and *Yucca gloriosa* L. [22,23,27,42]. It is worth noting that, within these 2 species, phytochemicals endemic to the genus have been found. Some of these have shown a particular effect against inflammation and oxidative stress [31]. Therefore, it could be expected to find other phytochemicals with similar structures among the other species. It has been reported that metabolomics can be used in taxonomical classifications [251]. If there are molecules that have a similar structure, it is very possible that they have a quite similar effect. This is based on the similarity principle, where similar molecules exhibit similar biological activity [252]. Thus, within the 50 species, there could be molecules with greater anti-inflammatory and antioxidant potential. Thus, there are unexplored unknowns related to most *Yucca* species. More specifically, to its anti-inflammatory and antioxidant properties and phytochemicals.

However, even with the favorable results of the research done with *Yucca schidigera* Roezl ex Ortgies and *Yucca gloriosa* L., further study of its use against inflammatory diseases is still needed. In particular, in vitro studies rarely cope with the complexity of human diseases [253]. From what could be found in the literature, only a few disease models have been used to study the therapeutic potential of *Yucca*, such as ovalbumin-induced airway hyperresponsiveness in mice [27]. Similarly, the efficacy and potency of different *Yucca* extracts have not been compared. Nor has its effect been compared with that of known treatments. Therefore, the study of *Yucca* genus against established models of inflammatory diseases is another field without much exploration. Finally, since the discovery of Yuccaol C and its mechanism of action against NO synthesis [31]. There has not been much research done on this topic. This is surprising since it is found in relatively large proportions within *Yucca schidigera* Roezl ex Ortgies and *Yucca gloriosa* L., as can be seen in Table 1. In addition to its reported efficiency, against the NF-kB pathway [31]. Thus, there is another unexplored *Yucca* topic. For the same reason, although there is favorable evidence on the anti-inflammatory and antioxidant capacity of *Yucca* genus. There is still a lot of research to be done before being able to describe the genus or its phytochemicals as an alternative to treat inflammatory diseases.

## 7. Conclusions

*Yucca* genus encompasses about 40 to 50 species, natives of southern North America. For centuries, it has been used to treat pathologies such as asthma, rheumatism, gonorrhea, sunburns, arthritis, etc. The ethnobotanical use led to the testing of many biological activities, where its antioxidant and anti-inflammatory excels. Unfortunately, there are a limited number of studies, so knowing its composition will provide a better understanding of the molecules responsible for these properties. This is because it is known that the medicinal use of plants is due to its phytochemicals. The documentary research found 92 phytochemicals with reported antioxidant and anti-inflammatory activities. Most of these molecules can be classified as phenolic compounds, glycosides, saponins, or fatty acids. Within these molecules, phytochemicals, such as Yuccaol C, stand out because they are original to the genus and have significant anti-inflammatory and antioxidant activity. The antioxidant and anti-inflammatory properties are mainly generated through free radical scavenging activity, the inhibition of arachidonic acid metabolism, the inhibition of MAPK and NF-κB, and the activation of Nrf2 signaling pathway. The NF-kB pathway is mainly inhibited by phytochemicals through the inhibition of IKK activation and phosphorylation IκBα, and the decrease of NF-kB concentration. The MAPK pathways are mainly inhibited by reducing p38, JNK, and ERK1/2 phosphorylation. Nrf2 is activated by increasing its concentration or inhibiting Keap1. However, there is evidence of the antioxidant and anti-inflammatory activity of some species within the genus, and although it is not abundant, the fact that a great variety of the phytochemicals that compose it present the same activities allows us to assess these properties.

## Figures and Tables

**Table 1:** Some of the antioxidant and anti-inflammatory effects of the phenolic compounds of the *Yucca* genus reported in the literature.

Metabolite	Species Where It Has Been Founded	Effect	Administrated Doses	References	
				Effect	Metabolite Screening
Resveratrol	*Yucca schidigera Yucca gloriosa* *Yucca periculosa* *Yucca elephantipes*	A Resveratrol supplement significantly reduced the concentrations of MDA and increased the levels of GPx, CAT, and SOD in horses	Horses were fed with 30 g (2 g of resveratrol) for 10 days and then 15 g (1 g of resveratrol) for 18 days.	[76]	[67,77,78,79]
		Resveratrol increased the expression of SIRT1 (Sirtuin 1) and decreased ROS and p38 levels in human umbilical endothelial cells with TNF-α-induced injury. By inhibiting the MAPK/NF-κB pathway.	The cells were treated with doses of 0, 5, 10, and 20 μM.	[80]	
		Resveratrol inhibited PGE2 (Prostaglandin E2) synthesis in murine embryonic fibroblasts ex vivo. This was found to be due to resveratrol’s ability to bind directly with COX-2.	The IC50 (Half maximal inhibitory concentration) was 60 μM.	[81]	
4,4’-Dihydroxystilbene (DHS)	*Yucca periculosa*	DHS decreased levels of MDA and 8-oxo-dG (8-Oxo-2’-deoxyguanosine), TNF-α, COX-2, MMP-9 (Matrix metalloproteinase 9), and p65 in mice with cigarette smoke-induced lung failure. It also activates Nrf2 by suppressing its ubiquitylation.	Dose of 2 and 4 mg/kg.	[82]	[77]
		DHS showed good antioxidant activity in vitro against the galvinoxyl radical, Cu (II) ions, and Fe2+/VC-induced lipid peroxidation in human erythrocyte ghosts.	Doses of 1, 2, and 3 μM were used for Fe^2+^/VC-induced lipid peroxidation.	[83]	
3,3’,5,5’-tetrahydroxy-4-methoxystilbene	*Yucca periculosa* *Yucca schidigera* *Yucca gloriosa*	The TEAC assay showed that 3,3’,5,5’-tetrahydroxy-4-methoxystilbene has the highest scavenging activity of all phenols in *Yucca schidigera*. It also reduced the effects of oxidative stress on blood platelets.	TEAC assay value was 2.252 mM	[67]	[67,77,78]
		It inhibited the carbonylation, nitration, and oxidation of proteins. Reduced peroxynitrite-induced platelet lipid peroxidation.	A concentration of 0.1 mM was used	[84]	
Yuccaol A	*Yucca schidigera Yucca gloriosa*	Yuccaol A significantly decreases the NO release in LPS-stimulated J774.A1 macrophages.	At a concentration of 100 µM	[34,78]	
Yuccaol C	*Yucca schidigera Yucca gloriosa*	Yuccaol C reduces iNOS expression by inhibiting the transcription factor NF-kB.	From 0.1 µM it reduces the expression of iNOS in J774.A1 macrophages induced by LPS (Lipopolysaccharide).	[34,78]	
Gloriosaol A	*Yucca gloriosa*	Showed an Antioxidant Activity higher than quercetin with the TEAC assay.	TEAC assay value was 5.55 mM	[69,78]	
Gloriosaol B	*Yucca gloriosa*	Showed an Antioxidant Activity higher than quercetin with the TEAC assay.	TEAC assay value was 3 mM	[69,78]	
Gloriosaol C	*Yucca gloriosa*	Showed an Antioxidant Activity higher than quercetin with the TEAC assay.	TEAC assay value was 5.6 mM	[69,78]	
Gloriosaol D	*Yucca gloriosa*	A combination of Gloriosaol D and E Showed an Antioxidant Activity higher than quercetin with the TEAC assay.	TEAC assay value was 4.91 mM	[69,78]	
Gloriosaol E	*Yucca gloriosa*				
Gallic acid	*Yucca elephantipes Yucca aloifolia variegata Yucca aloifolia*	Gallic acid increased Nrf2 expression, which suppresses ROS and IL-1β levels and blocked the activation of the inflammasome NLRP3 (Nucleotide-binding oligomerization domain-like receptor containing domain 3 of pyrin) in mice.	A dose of 100 mg/kg was injected intra-articular.	[85]	[86,87]
		Gallic acid significantly inhibited inflammation in a rat model of arthritis in a dose-dependent manner. This could be due to an inhibition of PDE4 (Phosphodiesterase 4) activity, and a decrease in TNF-α concentration.	Doses of 1, 5, and 10 μg/g.	[88]	
Chlorogenic acid	*Yucca elephantipes Yucca aloifolia variegata Yucca aloifolia*	Chlorogenic acid inhibited the production of TNF-α in and attenuated arthritis progression in collagen-induced arthritis mice. All of these are probably possible through the suppression of NF-κB pathways.	Doses of 30 mg/kg and 60 mg/kg for the attenuation of arthritis.	[89]	[79,86,87]
Cinnamic acid	*Yucca elephantipes Yucca aloifolia variegata Yucca aloifolia*	Cinnamic acid reduced the concentrations of MDA, TNF-α, and increased CAT activity in cisplatin-induced splenotoxicity in rats.	A dose of 50 mg/kg per 7 days	[90]	[86,87]
Hesperetin	*Yucca elephantipes* *Yucca aloifolia variegata*	Hesperetin decreased the levels of ROS, LPO (Lipid Peroxidation), TLR4 Toll-like receptor 4, p-NF-κB, and TNF-α and IL-1β, and increased the level of Nrf2 and HO-1 in mice with Aβ-induced neurodegeneration.	Doses of 50 mg/kg were injected for 6 weeks	[91]	[79,86,87]
		Hesperetin decreased the expression of NO, IL-1β, IL-6, and TNF-α in LPS-stimulated BV-2 microglial cells. This is done by inhibiting the activation of the ERK pathway.	Cells were pretreated with doses of 0, 5, 25, 50, 100, and 200 µM	[92]	
Naringenin	*Yucca elephantipes* *Yucca aloifolia variegata*	Mice with induced rheumatoid arthritis treated with naringenin significantly reduced inflammation and arthritis. It also decreased MPO and NO levels even more than those treated with methotrexate, a common treatment for arthritis.	The treatment against Rheumatoid Arthritis lasted three weeks with a dose of 40 mg/kg daily and orally.	[93]	[79,86,87]
		Naringenin pretreatment reduced the concentration of NO, PGE2, iNOS, COX-2, IL-1β, TNF-α, and MCP-1 (Monocyte chemoattractant protein 1) in BV2 microglial cells treated with LPS. Attenuation could be due to the suppression of NF-κB and MAPK pathways.	Cells were treated with 80 µM	[94]	
Luteolin	*Yucca elephantipes* *Yucca aloifolia variegata*	A clinical trial with a dietary formulation containing luteolin was administered to children with autism spectrum disorders (ASD) and it was found that this formulation significantly decreased IL-6 and TNF-α.	The dietary formulation contained 100 mg of luteolin, 70 mg of quercetin, and 30 mg of quercetin glycoside rutin, for 26 weeks.	[95]	[79,86,87]
		Luteolin decreased MPO, H_2_O_2_, MDA, and NF-κB levels, while increasing GPx, SOD, CAT activity, and GSH (Glutathione), and vitamin C levels in rats exposed to sodium fluoride.	Doses of 100 and 200 mg/kg/day orally for one week.	[96]	
Caffeic acid	*Yucca elephantipes* *Yucca aloifolia variegata*	Treatment with caffeic acid decreased the expression of NF-κB and IL-1β in Wistar rats with hearing loss	A dose of 30 mg/kg	[97]	[79,86,87]
		Caffeic acid showed effective antioxidant properties in vitro compared to standard antioxidants.	At 10 and 30 μg/mL, caffeic acid showed 68.2 and 75.8% inhibition of lipid peroxidation.	[98]	
Acacetin	*Yucca elephantipes* *Yucca aloifolia variegata*	Acacetin blocked the activation of NF-κB, decreased the activity of MPO, the expression of iNOS and COX-2, and increased the expression of SOD and HO-1 in sepsis-induced Acute lung injury mice.	Oral administration of 80 mg/kg showed the best results.	[99]	[86,87]
Epicatechin	*Yucca elephantipes*	Epicatechin decreased the levels of TNF-a, IL-6, NO, MPO, and MDA and inhibited NF-kB activation in mice with induced ulcerative colitis.	Doses of 100, 200, or 300 mg/kg were administered orally.	[100]	[79]
		Epicatechin has peroxyl radical scavenging activity.	At a concentration of 20.53 µmol.	[101]	
Protocatechuic acid	*Yucca elephantipes*	Protocatechuic acid showed higher antioxidant activity than Trolox in DPPH, ABTS, reducing power (Fe^3+^), reducing power (Cu^2+^), superoxide anion radical scavenging, hydroxyl radical scavenging, chelating capacity (Fe^2+^), and chelating capacity (Cu^2+^).	The ratio of IC50 Trolox/Protocatechuic acid was: 2.8, 2.3, 3.7, 6.1, 4.2, 1.0, 2.7, and 1.5, respectively.	[102]	[79]
		Protocatechial acid treatment decreased the levels of NO and LPO and increased GSH, CAT, and SOD in different rat models.	Doses of 25, 50, and 100 mg/kg orally	[103]	
Shikimic acid	*Yucca carnerosana* *Yucca elephantipes*	Shikimic acid treatment decreased TNF-α and IL-1β levels and inhibited of ERK 1/2 and p38 phosphorylation in LPS-stimulated RAW 264.7 macrophages.	Cells were cultured with 10 mM	[104]	[79,105]
Eriodictyol	*Yucca carnerosana*	Eriodictyol decreased NO production, prevented induced oxidation, and increased CAT activity in murine macrophages.	The mean effective concentrations of antioxidant activity were 14 μM.	[106]	[105]
		Pre-treatment with eriodictyol induced the Nrf2 pathway in mice with acute lung injury. This decreased the levels of TNF-α, IL-6, IL-1β, MIP-2 (Macrophage inflammatory protein 2), H_2_O_2_, and MDA	30 mg/kg was given orally 2 days before the acute lung injury.	[107]	
Scopoletin	*Yucca elephantipes*	Scopoletin had scavenging activity against superoxide anion in the xanthine/xanthine oxidase reaction system.	At concentration of 12.5, 25, 50, and 100 µm.	[108]	[79]
		Scopoletin reduced PGE2 and TNF-α expression, MPO activity, and MDA levels in carrageenan-induced mice.	A dose of 100 mg/kg	[109]	
Nordihydrocapsiate	*Yucca carnerosana*	Nordihydrocapsiate inhibits NF-κB activation by inhibiting IκBα degradation and IKK activation.	At a concentration of 100 μM	[110]	[105]
Myricetin	*Yucca elephantipes*	Myricetin protects cells against H_2_O_2_-induced cell damage by scavenging radicals and restoring the expression of SOD, CAT, and GPx.	At a concentration of 10μg/ml	[111]	[79]
		Myricetin suppresses activation of the NF-κB pathway; by inhibiting AKT and NF-κB p65 phosphorylation; and the MAPK signaling pathway; by inhibiting the phosphorylation of JNK, p-ERK, and p38.	At a concentration of 12.5 and 25 μM	[112]	
Ellagic acid	*Yucca elephantipes*	Ellagic acid showed high radical scavenging capacity against DPPH, inhibits lipid peroxidation, and increases SOD, CAT, and GPX activity in V79-4 cells.	4, 20, and 100 µg/ml	[113]	[79]
		Ellagic acid intake decreased the expression of IL-1beta, IL-6, TNF-α, and MCP-1 and increased that of GPx, SOD, and CAT in diabetic mice.	Ellagic acid at 2%	[114]	
Nordihydroguaiaretic acid	*Yucca elephantipes*	Nordihydroguaiaretic acid activates the Nrf2/HO-1 antioxidant pathway in cerebellar granule neurons.	At a concentration of 20 µM	[115]	[79]
		Nordihydroguaiaretic acid suppressed ERK activation in RANKL-treated mouse RAW-D and BMM cells.	At a concentration of 5 µM	[116]	
Sinapic acid	*Yucca carnerosana* *Yucca elephantipes*	Sinapic acid has a good antioxidant capacity in vitro.	It had an IC50 of 32.2 μM in the DPPH and for the Ferric Reducing Antioxidant Power assay of 482.6 μM/mol	[117]	[79,105]
		Sinapic acid inhibited the expression of iNOS, COX-2, TNF-α, and IL-1β in RAW 264.7 cells induced with LPS. These observations are due to the suppression of the nuclear translocation of the p65 and p50 NF-κB subunits and the degradation of IκBα.	The reported inhibition was with pretreatment of 30 mg/kg orally 1 h before the induction of paw edema.	[118]	
Cirsimarin	*Yucca elephantipes*	Cirsimarin in vitro inhibited lipid peroxidation and induced a 36% reduction in H_2_O_2_ production in adipose tissue.	IC50 = 370 µM in lipid peroxidation and 15 µM in H_2_O_2_ production.	[119]	[79]
Rosmarinic acid	*Yucca elephantipes* *Yucca aloifolia variegata*	Rosmarinic acid reduced the levels of MPO, NO, IL-6, IL-1β, TNF-α, iNOS, and COX-2 in mice with dextran sodium sulfate-induced colitis.	The dose range of 25–100 mg/kg	[120]	[79,87]
		Rosmarinic acid showed protective activity against peroxynitrite-mediated damage and inhibited NO and iNOS in RAW264.7. This is done by inhibiting the serine phosphorylation of IκBα.	At a concentration of 10, 50 mm	[121]	
		Rosmarinic increased the activity of SOD, CAT, and GPx in aged mice.	Results were seen at a dose of 200 mg/kg once daily for 30 days.	[122]	
Psoralen	*Yucca elephantipes*	Psoralens inhibit the expression of TNF-α, IL-1β, IL-6, and IL-8, and proteins involved in the TLR4-IRAK4-NF-κb pathway in LPS-stimulated cells. The anti-inflammatory activity could be due to an agonist effect on the estrogen receptor.	At a concentration of 12.5 micrograms/mL	[123]	[79]
		Psoralen inhibits the expression of MMP-1, -2, -3, -9, -12, -13 and IL-1β, -6, -12 in human synoviocytes cultured with TNF-α.	At a concentration of 1,10 and 20 µM	[124]	
4-Hydroxyphenylacetic acid	*Yucca elephantipes*	4-Hydroxyphenylacetic acid increased Nrf2 translocation to the nucleus, which enhanced antioxidant enzymes in mice with induced acute liver failure.	The mice were treated with 4-hydroxyphenylacetic acid at doses of 6, 12, or 25 mg/kg for 3 days.	[120]	[79]
Angelicin	*Yucca elephantipes*	Angelicin inhibited IL-4, IL-5, and IL-13 production and NF-kB activation in mice with ovalbumin-induced asthma.	Pretreatment of 2.5, 5, and 10 mg/kg was administered intraperitoneally	[125]	[79]
		Angelicin blocked the phosphorylation of IκB-α, NF-κBp65, p38 MAPK, and JNK in mice with LPS-induced acute lung injury.	Doses of 1, 5, and 10 mg/kg were injected intraperitoneally.	[126]	
Ferulic acid	*Yucca carnerosana* *Yucca elephantipes* *Yucca aloifolia variegata*	Ferulic acid demonstrated an anti-inflammatory effect and reduced IL-1β and TNF-α levels in mice with collagen-induced arthritis.	1.28 μg/g/d were administered intragastrically twice a day.	[107]	[79,87,105]
		Ferulic acid reduced superoxide radicals and ICAM-1 (Intercellular adhesion molecule) and NF-κB expression in mice with cerebral artery occlusion.	Dose of 100 mg/kg, intravenously	[127]	
6-Methylcoumarin	*Yucca aloifolia variegata*	6-methylcoumarin reduced NO, PGE2, iNOS, COX-2 levels, and MAPK phosphorylation and degradation of RAW 264.7 cells stimulated by IκB-α LPS	Cells were pretreated with doses of 300, 400, and 500 μM for 1 h	[128]	[87]
Citropten	*Yucca elephantipes*	Citropten Reduces NFκB and MAPK Signaling Pathway, Alleviates Colon Inflammation and Effector T Cell Activity in Mice with DSS-Induced Colitis.	Oral administration of 10 mg/kg and 40 mg/kg	[129]	[79]
Emodin	*Yucca elephantipes*	Emodin decreased levels of amylase, lipase, TNF-α, IL-18, and ROS in rats with severe acute pancreatitis.	At a concentration of 6 mg/mL	[130]	[79]
		Emodin reduced the levels of TNF-α, IL-6, MPO, MDA, Cytokine-induced neutrophil chemoattractant, MIP-2, ICAM-1, and NF-κB nuclear translocation in cholestatic-induced hepatitis rats.	20 mg/kg/d were administered intragastrically for 4 days.	[131]	
Chrysin	*Yucca elephantipes*	Chrysin treatment inhibits p65 NF-κB unit, TNF-α, IL-1β, and IL-6 levels, where 100 mg/kg had the same effect as 100 mg/kg methylprednisolone in rats with spinal cord injury. It also reduced the concentration of iNOS and NO.	Doses of 30 and 100 mg/kg/day for 26 days.	[132]	[79]
		Chrysin treatment decreased lipid peroxidation and increased the activities of SOD, CAT GPx, GSH, vitamin C, and vitamin E in hepatitis-induced rats.	Doses of 25, 50, and 100 mg/kg/day orally for 6 days.	[133]	
Alpinetin	*Yucca carnerosana*	Alpinetin decreased levels of ROS, MDA, NLRP3 inflammasome activation, and IL-1β, IL-6, TNF-α, COX-2, and iNOS in mice with induced liver fibrosis. This could be because the nuclear expression of Nrf2 increased.	Mice were injected intraperitoneally with doses of 15 and 60 mg/kg.	[134]	[105]
		Alpinetin repressed p65 nuclear translocation, and IκB phosphorylation, and stimulated ERK phosphorylation in TNF-α-stimulated rat chondrocytes for 48 h.	The chondrocytes were treated with doses of 10 μM and 20 μM,	[135]	
Vanillin	*Yucca elephantipes*	Vanillin treatment in ICR mice increased plasma antioxidant activity as demonstrated by the ORAC assay. It also showed good antioxidant capacity in vitro ith: DPPH radical, galvinoxyl radical, ABTS• scavenging assays, and Oxidative Hemolysis Inhibition Assay.	Doses of 25 mg/mL dissolved were administered orally.	[136]	[79]
		Vanillin pretreatment inhibited hepatic lipid peroxidation, protein carbonyl formation, expression of TNF-α, IL-6, IL-1β, and increased CAT, SOD, and GSH levels in rats with induced hepatotoxicity.	Doses of 150 mg/kg were injected intraperitoneally for 3 days before induction of hepatotoxicity.	[137]	
Quercetin	*Yucca aloifolia variegata* *Yucca elephantipes*	Quercetin pretreatment decreased the expression of NF-κB, VCAM-1, ICAM-1, E-selectin, and AP-1 in human umbilical vein endothelial cells induced with TNF-α.	The cells were cultured with quercetin for 18 h at a dose of 30 μg/mL.	[138]	[79,87]
		Quercetin significantly increased CAT, SOD, and GPx activity in rats with induced diabetes.	A dose of 15 mg/kg/day was injected intraperitoneally.	[139]	
		Quercetin decreased IL-1β, TNF-a levels, and IκBα expression in patients with coronary artery disease.	A daily dose of 120 mg for 2 months.	[140]	
Kaempferide	*Yucca elephantipes*	Kaempferol decreased levels of TNF-α, iNOS, IL-12, p38, and IκBα phosphorylation, p65 nuclear translocation, and increased HO-1 and Nrf2 levels in mice with nephrotoxicity.	Doses of 50, 100, 150 and 200 mg/kg.	[141]	[79]
Procyanidin B2	*Yucca aloifolia variegata* *Yucca elephantipes*	Procyanidin B2 decreased the expression of p65, COX2, iNOS, IL-6, IL-1β, NO production, and blocked NLRP3 inflammasome activation in LPS-treated THP-1 macrophages.	Cells were treated with 10 μM.	[142]	[79,87]
		Procyanidin B2 decreased PSA, TNF-α, IL-6, and IL-10 levels and increased SOD, CAT, GSH, Nrf2, and HO-1 levels in rats with chronic induced prostatitis.	Doses of 20 and 40 mg/kg were administered for 4 weeks.	[143]	
Umbelliferone	*Yucca elephantipes*	Umbelliferone decreased lipid peroxidation and increased vitamin C, vitamin E, GSH levels, and SOD, CAT, and GPx activity in rats with induced diabetes.	Dose of 30 mg/kg for 45 days.	[144]	[79]
		Umbelliferone reduced the levels of MPO, MDA, COX-2, PGE2, NF-kB, VEGF (Vascular endothelial growth factor), TNF-α, IL-1β, IL-6, IL-10, and IL-17 in rats treated with complete Freund’s adjuvant.	Doses of 10, 20, 40 mg/kg for 28 days	[145]	
Catechin	*Yucca elephantipes*	Catechin decreased NO production and iNOS, COX2, TNF-α, and IL-1β expression in LPS-stimulated RAW 264.7 cells. Combined treatment with quercetin and catechin was also shown to have synergistic anti-inflammatory effects.	At a concentration of 150μM	[146]	[79]
		Catechin decreased MDA level and increased SOD, GST (Glutathione-S-transferase), and CAT activities in diabetic-induced rats.	Doses of 20, 40 or 80 mg/kg/day	[147]	
Scopolin	*Yucca elephantipes*	Scopolin reduced IL-6, VEGF, and FGF-2 in rats with adjuvant-induced arthritis.	Doses of 50 or 100 mg/kg were injected intraperitoneally for 10 days	[148]	[79]
Kaempferol	*Yucca aloifolia variegata* *Yucca elephantipes*	Kaempferol reduced iNOS, COX-2, and CRP (Reactive C-protein) expression and inhibits NF-κB activation by inhibiting IκBα degradation and IKK activation in the human hepatocyte-derived Chang Liver cell line.	Concentration from 5 to 200 μmol/L	[149]	[79,87]
		Kaempferol pretreatment decreased ROS excess and increased SOD, CAT, and GPx levels in human erythrocytes with ROS-induced hemolysis.	A dose of 100 μg/mL suppressed 87.4% of hemolysis	[150]	
Hesperidin	*Yucca elephantipes* *Yucca aloifolia variegata Yucca aloifolia Yucca flamentosa*	Hesperidin decreased the levels of MDA, NO, NF-kB and p-Akt, while it increased the levels of GSH in rats with acute cisplatin-induced hepatotoxicity.	Dose of 100 or 200 mg/kg/day for 7 days	[151]	[79,87]
		Hesperidin showed good scavenging potential against DPPH and protected yeast cells against stressors.	25 and 50 µM were used in all tests	[152]	
Apigenin	*Yucca elephantipes* *Yucca aloifolia variegata*	Apigenin pretreatment decreased NF-kB and COX-2 expression, and IL-6, IL-1β, and TNF-α levels in ALI mice with LPS-induced inflammation.	Doses of 10 or 20 mg/kg were injected intraperitoneally.	[153]	[79,87]
		Apigenin decreased LPO levels and increased SOD, CAT, GPx, vitamin C, vitamin E, and GSH levels in rats with oxidative stress caused by the carcinogen.	A dose of 25 mg/kg/day was administered intraperitoneally for 14 days.	[154]	
Vanillic acid	*Yucca elephantipes*	Vanillic acid pretreatment inhibited oxidative stress, IL1β, TNF-α, and IL-33 expression and suppressed NF-κB activation in inflammation-induced mice.	Doses of 3–30 mg/kg injected intraperitoneally.	[155]	[79]
		Vanillic acid pretreatment reduced lipid peroxidation and decreased IL-1β, IL-6, and TNF-α expression in rats with induced cardiotoxicity. It also showed good radical scavenging ability in vitro.	Dose of 5 mg or 10 mg/kg/day for 10 days.	[156]	

Table 1 abbreviations: MDA = Malondialdehyde, GPx = Glutathione peroxidase, CAT = Catalase, SOD = Superoxide dismutase, SIRT1 = Sirtuin 1, ROS = Reactive oxygen species, MAPK = Mitogen-Activated Protein Kinase, NF-κB = Nuclear transcription factor-κB, TNF-α = Tumor necrosis factor α, PGE2 = Prostaglandin E2, COX-2 = Cyclooxygenase-2, IC50 = Half maximal inhibitory concentration, 8-oxo-dG = 8-Oxo-2’-deoxyguanosine, MMP-9 = Matrix metalloproteinase-9, Nrf2 = Nuclear factor erythroid 2–related factor 2, TEAC = Trolox equivalent antioxidant capacity, NLRP3 = Nucleotide-binding oligomerization domain-like receptor containing domain 3 of pyrin, PDE4 = Phosphodiesterase 4, IL = Interleukin, NO = Nitric oxide, ERK = Extracellular signal-regulated kinase, LPO = Lipid Peroxidation, TLR4 = Toll-like receptor 4, iNOS = Inducible nitric oxide synthase, MCP-1 = Monocyte chemoattractant protein-1, MPO = Myeloperoxidase, PCO = Protein carbonyl, GSH = Glutathione, Kim-1 = Kidney injury marker 1, CTnI = Cardiac troponin I, ALI = Acute lung injury, HO- 1 = Heme oxygenase 1, IKB-α = Inhibitor of nuclear factor kappa B, LPS = Lipopolysaccharide, MIP-2 = Macrophage inflammatory protein-2, IKK = IkB kinase, JNK = Jun N-terminal kinase, IRAK-4 = Interleukin-1 receptor-associated kinase 4, ICAM-1 = Intercellular adhesion molecule, CINC-1 = Cytokine-induced neutrophil chemoattractant, VEGF = Vascular endothelial growth factor, GST = Glutathione-S-transferase, FGF2 = Fibroblast growth factor 2, CRP = Reactive C-protein.

**Table 2:** Some of the antioxidant and anti-inflammatory effects of saponins of the *Yucca* genus reported in the literature.

Metabolite	Species Where It Has Been Founded	Effect	Administrated Doses	References	
				Effect	Metabolite Screening
Sarsasapogenin	*Yucca schidigera* *Yucca aloifolia variegata*	Sarsasapogenin suppressed NLRP3 inflammasome activation and the NF-κB pathway by downregulating PAR-1 (Protease-activated receptor 1) expression in rats with induced diabetes.	Dose 20 or 60 mg/kg/day by intragastric tube for 10 weeks.	[163]	[87]
		Sarsapogenin decreases the levels of MDA, MPO, NO, TNF-α, IL-12, IL-6, TXB2 (Thromboxane B2), and IgE (Immunoglobulin E) in mice with induced asthma.	A dose of 50 µg/mouse	[164]	
Hecogenin	*Yucca elephantipes Yucca aloifolia variegata Yucca glauca*	Hecogenin decreased NF-κB and p53 expression levels, decreased lipid peroxidation, and increased SOD, GPx, GST, CAT, vitamin E, vitamin C, and GSH levels in rats with induced myocardial infarction.	50 µg/kg dose orally for 21 days	[165]	[86,87]
		Hecogenin reduced the levels of MDA, MPO, TNF-α, IL-6, IL12, TXB2, IgE and NO, and increased SOD, GSH in rats with induced colitis	Dose of 50 µg/rat transrectally at 72, 48, 24 and 2 h before colitis induction.	[166]	
		Hecogenin decreases the levels of MPO, IL-6, IL-2, TBX2, and COX-2 expression in rats treated with complete Freund’s adjuvant.	At a concentration of 50 µg/kg dose, orally	[167]	
Diosgenin	*Yucca aloifolia variegata*	Diosgenin increased SOD, GPx levels, and decreased ROS concentration, TBARS (Thiobarbituric acid-reactive substances), caspase-3 activation, NF-κB expression, and modulated PKA (Protein kinase A) and p38 activation.	Oral feeding at a dose of 130 mg/kg daily for 4 weeks.	[168]	[87]
Timosaponin BII	*Yucca glauca*	Timosaponin BII decreased IL-6, TNF-α, MDA levels and increased SOD, HO-1 and Nrf-2 levels in rats with induced myocardial infarction.	Dose of 50 or 100 mg/kg/day by gastric tube for 5 days.	[169]	[170]
		Timosaponin BII decreased phosphorylation of mTOR (Mammalian target of rapamycin) and NF-κB.	100, 300, and 500 mg/kg/day orally for 3 months	[171]	
Timosaponin AIII	*Yucca gloriosa* *Yucca macrocarpa*	Thymosaponin AIII decreased IL-1β, TNF-α, IL-6, MPO levels, NF-κB activation, and increased IL-10 concentration in mice with induced colonic shortening. It also inhibited the binding of LPS to the Toll-like receptor 4 of macrophages and could be metabolized to sarsasapogenin by intestinal microbiota, a molecule that has also shown anti-inflammatory activity.	Oral administration of 5 or 10 mg/kg/day for 3 days.	[172]	[170]
Degalactotigonin	*Yucca gloriosa*	Degalactotigonin inhibited the phosphorylation of the EGFR (Epidermal growth factor receptor) in human pancreatic cancer cell lines PANC1.	At a concentration of 100 ng/mL for 24 h	[173]	[78,170]
Sweroside	*Yucca aloifolia variegata*	Sweroside pretreatment inhibited Keap1, induced Nrf2 nuclear translocation, inhibited ROS, and increased SOD and GPx activity in hypoxia/reoxygenation-induced H9c2 cells.	At a concentration of 50 μM for 24 h	[174]	[87]
		Sweroside activated SIRT1, suppressed NF-κB, and promoted Forkhead O1 transcription factor signaling pathways in LPS-induced RAW264.7 cells. 20,	At a concentration of 40 and 80 µM	[175]	

Table 2 abbreviations: NLRP3 = Nucleotide-binding oligomerization domain-like receptor containing domain 3 of pyrin, NF-κB = Nuclear transcription factor-κB, PAR-1 = Protease-activated receptor 1, MDA = Malondialdehyde, GPx = Glutathione peroxidase, CAT = Catalase, SOD = Superoxide dismutase, MPO = Myeloperoxidase, NO = Nitric oxide, TNF-α = Tumor necrosis factor α IL = Interleukin, TXB2 = Thromboxane B2, IgE = Immunoglobulin E, GST = Glutathione-S-transferase, GSH = Glutathione, COX-2 = Cyclooxygenase-2, TBARS = Thiobarbituric acid-reactive substances, PKA = Protein kinase A, ROS = Reactive oxygen species, HO- 1 = Heme oxygenase 1, Nrf2 = Nuclear factor erythroid 2–related factor, mTOR = Mammalian target of rapamycin, LPS = Lipopolysaccharide.

**Table 3:** Some of the antioxidant and anti-inflammatory effects of Glycosides of the *Yucca* genus reported in the literature.

Metabolite	Species Where It Has Been Founded	Effect	Administrated Doses	References	
				Effect	Metabolite Screening
Vitexin-2″-O-rhamnoside	*Yucca elephantipes*	Vitexin-2″-O-rhamnoside had an antioxidant effect against tert-butyl-hydroperoxide-induced lipid peroxidation in ECV304 cell culture.	The optimal concentration in ECV304 cell culture was 62.5 μmol/L	[180]	[86]
Isosakuranetin-7-O-neohesperidoside (Poncirin)	*Yucca elephantipes*	A treatment with poncirin showed improvement in the liver in male albino mice with Carbon tetra chloride-induced liver injury. This is done by neutralizing nitric oxide production, enhancing the concentration of GST, GSH, CAT, SOD, and attenuating the activity of lipid peroxidase, MPO, and the levels of IL-1β, IL-6 and TNF-α.	At a concentration of 30 mg/kg.	[181]	[86]
		Poncirin improved symptoms associated in mice with liver injury induced by paracetamol. This is done by decreasing the expression of NF-κB, JNK, and COX-2.	At a concentration of 30 mg/kg.	[182]	
Apigenin-7-O-β-d-glucoside	*Yucca elephantipes*	Apigenin-7-O-β-d-glucoside showed antioxidant activity in vitro.	Where its IC50 (μg/mL) for ABTS was 36.5 ± 0.76, O•2− 86.4 ± 0.21, DPPH• 34.2 ± 0.98 and •OH 155.2 ± 0.25.	[100]	[86]
Myricitrin	*Yucca elephantipes*	Myricitrin treatment increased GSH level and Cytochrome P450 2E1 expression, and decreased lipid peroxidation, and COX-2 and TNF-a expression in mice with tetrachloride intoxication. carbon (CCl4)	Doses of 10, 30 and 100 mg/kg by oral tube, once a day for 2 days after poisoning.	[183]	[79]
		Myricitrin can effectively scavenge free radicals in vitro and decrease acrylamide toxicity by inhibiting ROS generation in human gastrointestinal Caco-2 cells.	At a concentration of 2.5–10 μg	[184]	
Kaempferol dirhamnoside	*Yucca aloifolia variegata*	In vitro kaempferol dirhamnoside showed high reactivity with DPPH, inhibited MPO activity and decreased ascorbyl radical-induced lipid peroxidation.	The IC50s were: 84.0 ± 7.8 μM, 86 ± 9.9 μM, 320 ± 14.1 μM, respectively	[185]	[87]
Phloridzin	*Yucca elephantipes*	Phloridzin increased SOD, GSH levels, and decreased TNF-α, IL-6, and MDA levels in LPS-treated mice.	10–20 mg/kg, orally	[186]	[79]
Sweroside	*Yucca aloifolia variegata*	Sweroside pretreatment inhibited Keap1, induced Nrf2 nuclear translocation, inhibited ROS, and increased SOD and GPx activity in hypoxia/reoxygenation-induced H9c2 cells.	At a concentration of 50 μM for 24 h	[174]	[87]
		Sweroside activated SIRT1, suppressed NF-κB, and promoted Forkhead O1 transcription factor signaling pathways in LPS-induced RAW264.7 cells. 20,	At a concentration of 40 and 80 µM	[175]	
Kaempferol-3-O-glucoside	*Yucca elephantipes*	Kaempferol-3-O-glucoside had a good antioxidant activity with the DPPH radical scavenging method.	The mean IC50 of 1.25 ± 0.09 µg/mL	[187]	[79]
Quercitrin	*Yucca aloifolia variegata* *Yucca elephantipes*	Quercitrin decreased lipid peroxidative products and increased SOD, CAT, GPx, activity in rats with induced diabetes.	Oral administration of 30 mg/kg for 30 days	[188]	[79,87]
		Quercitrin decreased iNOS expression in rats with induced colitis. This was due to the inhibition of NF-κB activity.	Oral administration of 1 or 5 mg/kg/day	[189]	
		Quercetin reduced MPO activity, TNF-α and IL-1β expression, iNOS, and reduced NF-κB activation in rats with colitis.	Oral administration of 1 mg/kg/day	[190]	
Rutin	*Yucca aloifolia variegata* *Yucca elephantipes*	Rutin decreased MDA, ROS, NF-kBp65, TNF-α, and caspase-3 levels, as well as increased GSH levels, CAT activity, and Nrf-2 and HO-1 expression in rats with diabetic neuropathy.	Doses of 100 or 200 mg/kg were injected intraperitoneally for 8 weeks.	[191]	[79,87]
		Rutin showed good radical scavenging activity in DPPH assay, total antioxidant activity, reducing power, hydroxyl radical scavenging assay, superoxide radical scavenging assay and lipid peroxidation assay.	At the concentration of 0.05 mg/mL it showed an inhibition of 90.4% in DPPH and the IC50 was 24 mg/mL for the lipid peroxidation assay.	[192]	
Naringin	*Yucca aloifolia variegata* *Yucca elephantipes*	Naringenin decreased the levels of iNOS, ICAM-1, COX-2, MCP-1, TNF-α, TLR4, IL-6, p65, and p-IkBa in mice with induced colitis.	Dosage of 50 mg/kg/day orally for 10 days.	[193]	[79,87]
		Naringenin supplementation increased SOD and CAT activities in hypercholesterolemic patients.	400 mg/capsule/day for 8 weeks	[194]	
Hyperoside	*Yucca aloifolia variegata*	Hyperoside decreased levels of TNF-α, IL-6, NO, NF-κB, and IκB-α degradation in lipopolysaccharide-stimulated mouse peritoneal macrophages.	The maximum rate of inhibition by hyperoside 5 μM.	[195]	[87]
		Hyperoside decreased the levels of TNF-α, and the activation of AKT, NF-κB and ERK 1/2 in HUVEC.	Cells were treated with doses of 5, 10, 20 or 50 µM	[196]	
		Hyperoside scavenged ROS, which prevented lipid peroxidation, protein carbonyl, and cellular DNA damage in cells with H_2_O_2_-induced damage.	Cells were treated with 5 µM.	[197]	
Esculin	*Yucca aloifolia variegata*	Esculin decreased levels of TNF-α, IL-6, MDA, MCP-1, Intracellular adhesion molecule 1, and p38, and increased SOD activity in C57BL/6J mice.	Dose of 5, 10, or 20 mg/kg for 2 weeks.	[198]	[87]
		Esculin decreased NO, MPO, and MDA levels while increasing SOD activity in mice with acute gastric injury.	Doses of 12.5, 25 and 50 mg/kg	[199]	
Kaempferol-3-O-rhamnoside	*Yucca aloifolia variegata*	Kaempferol-3-O-rhamnoside decreased the levels of IL-4, IL-5 and IL-13, TNF-α, IgE, this through the inhibition of Akt phosphorylation.	Doses of 26 mg/kg/day were administered orally for 6 days.	[200]	[87]

Table 3 abbreviations: EGFR = Epidermal growth factor receptor, NF-κB = Nuclear transcription factor-κB, MDA = Malondialdehyde, GPx = Glutathione peroxidase, CAT = Catalase, SOD = Superoxide dismutase, MPO = Myeloperoxidase, NO = Nitric oxide, TNF-α = Tumor necrosis factor α IL = Interleukin, = Immunoglobulin E, GST = Glutathione-S-transferase, GSH = Glutathione, COX-2 = Cyclooxygenase-2, ROS = Reactive oxygen species, HO- 1 = Heme oxygenase 1, Nrf2 = Nuclear factor erythroid 2–related factor, LPS = Lipopolysaccharide, JNK = Jun N-terminal kinase, CYP2E1 = Cytochrome P450 2E1, Keap1 = Kelch-like ECH-associated protein 1, SIRT1 = Sirtuin 1, iNOS = Inducible nitric oxide synthase, MDA = Malondialdehyde, MPO = Myeloperoxidase, ICAM-1 = Intracellular adhesion molecule 1, MCP-1 = monocyte chemoattractant protein 1, TLR4 = Toll-like receptor 4, IKB-α = Inhibitor of nuclear factor kappa B, ERK = Extracellular signal-regulated kinase, IgE = Immunoglobulin.

**Table 4:** Some of the antioxidant and anti-inflammatory effects of fatty acids of the *Yucca* genus reported in the literature.

Metabolite	Species Where It Has Been Founded	Effect	Administrated Doses	References	
				Effect	Metabolite Screening
Gamma-Linolenic acid	*Yucca elephantipes* *Yucca aloifolia variegata*	Treatment with α-linolenic acid inhibited the production of PGE2 in on synovial membrane explant cultures with LPS-induced inflammation.	At a concentration of 300 µg/mL	[204]	[86,87]
		α-linolenic acid inhibited PGE2 production in Aβ-stimulated PC12 cells. It also suppressed IκB-α degradation, which blocked NF-κB activation.	At a concentration of 100 µM	[205]	
Caproic acid	*Yucca aloifolia variegata*	Capric acid suppressed MAPK phosphorylation and NF-kB activation in THP-1 cells treated with *P. acnes*.	100 μM	[206]	[87]
Lauric acid	*Yucca aloifolia variegata*	Lauric acid suppressed MAPK phosphorylation and NF-kB activation in THP-1 cells treated with *P. acnes*.	100 μM	[206]	[87]
		Lauric acid had inhibitory activity against COX-1 and COX-2	Inhibitory activity against COX-1 and COX-2 at 100 µg/mL.	[207]	
Pentadecanoic acid	*Yucca elephantipes* *Yucca aloifolia variegata*	Pentadecanoic acid decreased the levels of MCP-1 and IL-6 in C57BL/6 J mice	5 mg/kg for 12 weeks	[208]	[79,87]
Gama-linoleic acid	*Yucca aloifolia variegata*	Linoleic acid inhibited NO production and decreased p50 subunit expression of NF-κB, TNF-α, IL-6, IL-1β, and nitric oxide synthase 2 in LPS-treated RAW 264.7 cells. This could be due to the activation of the peroxisome proliferator-activated receptor-α.	150 µM	[209]	[21,87]
Stearic acid	*Yucca aloifolia variegata*	Stearic acid inhibits lipid peroxidation and increases SOD and CAT activity in cultured rat cortical neurons.	3,10 and 30 µmol/L	[210]	[21,87]
		Stearic acid attenuated NF-κB activation in rats with cholestasis and leukocyte accumulation.	Intraperitoneal administration of 1000 nmol/kg/day was started 2 weeks before injury and continued for 5 weeks.	[211]	
Oleic acid	*Yucca aloifolia variegata*	Oleic acid decreases the concentration of IL-1β, IL-6, and TNF-α and NF-κβ in rats with cadmium-induced lesions in heart and liver tissues.	10 mg/kg, orally	[212]	[21,87]
Cis-5,8,11,14,17-Eicosapentaenoic acid	*Yucca aloifolia variegata*	Pretreatment with 5,8,11,14,17-eicosapentaenoic acid decreased TNF-α expression by preventing IκB-α phosphorylation in LPS-induced THP-1 cells.	Cells were incubated with 60 μM	[213]	[87]
9,12-Octadecadienoic acid	*Yucca elephantipes*	9,12-octadecadienoic acid had antioxidant activity with an IC50 value of 45.65 µg/mL by the DPPH Radical Scavenging Method. Anti-inflammatory activity was also tested in rats with carrageenan-induced edema, where treatment with 9,12-Octadecadienoic acid showed a significant reduction in edema paw volume.	150μg/kg	[214]	[79]

Table 4 abbreviations: PGE2 = Prostaglandin E2, LPS = Lipopolysaccharide, IKB-α = Inhibitor of nuclear factor kappa B, NF-κB = Nuclear transcription factor-κB, MAPK = Mitogen-Activated Protein Kinase, COX-1 = Cyclooxygenase-1, COX-2 = Cyclooxygenase-2, MCP-1 = Monocyte chemoattractant protein 1, IL = interleukin, PPARα = Peroxisome proliferator-activated receptor-α, CAT = Catalase, SOD = Superoxide dismutase, TNF-α = Tumor necrosis factor α.

**Table 5:** Some of the antioxidant and anti-inflammatory effects of other phytochemicals of the *Yucca* genus reported in the literature.

Metabolite	Group	Species Where It Has Been Founded	Effect	Administrated Doses	References	
					Effect	Metabolite Screening
Stigmasterol	Phytosterol	*Yucca aloifolia variegata*	In chondrocytes from newborn mice or human osteoarthritis treated with IL-1β, preincubation with stigmasterol decreased the expression of Matrix Metallopeptidase 3, Matrix Metallopeptidase 13, ADAMTS-4 and PGE2, this by counteracting the effect of IL-1β on the NF-κB pathway.	Cells were pre-incubated for 48 h with 20 μg/ml	[218]	[87]
			Stigmasterol treatment improved clinical severity by reducing joint destruction and decreasing the expression of TNF-α, IL-6, IL-1β, iNOS and COX-2, p65 and p38 by inhibiting the activation of p- IκBα in collagen-induced arthritic rats.	200 mg/kg orally daily for 20 days	[219]	
			In rats with ischemia/reperfusion brain injury, stigmasterol treatment decreased COX-2 and p65 expressions. In addition to significantly increasing the expression of Nrf2, HO-1, SOD, CAT, and GPx.	Dosis de 20, 40 y 80 mg/kg	[220]	
Phytol	Terpenes	*Yucca aloifolia variegata*	Phytol treatment reduced MPO activity and the concentration of TNF-α, IL-6 and COX-2. By downregulating p38 and NF-κB signaling pathways in a mouse model of arthritis induced by complete Freund’s adjuvant.	Oral administration of 50 mg/kg	[221]	[87]
			Phytol exhibits a dose-dependent anti-inflammatory effect in formalin-induced paw edema by decreasing the levels of COX-1, COX-2, NF-κB and IL-1 β.	100 mg/kg of phytol	[222]	
			Phytol demonstrated a strong antioxidant effect in vitro. Eliminating hydroxyl radicals and nitric oxide and preventing the formation of TBARS	The concentration of 0.9, 1.8, 3.6, 5.4 and 7.2 ng/mL was used in the in vitro tests.	[223]	
Malic acid	Dicarboxylic acid	*Yucca aloifolia variegata*	Malic acid decreases TNF-α levels and inhibits platelet aggregation in rats with myocardial ischemia/reperfusion injury.	Doses of 500 mg/kg and 250 mg/kg	[224]	[87]
Loliolide	Terpenes	*Yucca aloifolia variegata*	Loliolide was found to exert dose-dependent positive effects on the protective effects against peroxide-induced cell damage.	Cells were pretreated with 250 and 500 μM of loliode	[225]	[87]
			Loliolide treatment in LPS-stimulated RAW 264.7 macrophages decreased TNF-α and IL-6 production.	100 µM	[226]	
			Loliolide treatment in LPS-stimulated RAW 264.7 macrophages downregulated IκBα and p65 in a dose-dependent manner, thereby inhibiting NF-κB nuclear translocation. In addition, it downregulates the proteins involved in the MAPK pathway (p38, ERK, and JNK), thus inhibiting MAPK phosphorylation.	15.6, 31.2, 62.5 µm/mL	[227]	
α-Tocopherol	Tocopherol	*Yucca aloifolia*	Oral α-tocopherol supplementation maintains cellular redox status in polychlorinated biphenyl-induced toxicity in rat liver, lung, and kidney. Therefore, the activities of SOD, CAT, GPx, and glutathione reductase are maintained, and the levels of lipid peroxides, hydroxyl radicals, and hydrogen peroxides are kept low.	50 mg/kg body weight/day	[228]	[21]
			α-Tocopherol treatment significantly reduced IL-6 levels, thereby inhibiting NF-κB-mediated gene transcription in cancerous mice.	150 mg/kg per day orally for 15 consecutive days	[229]	
γ-Tocotrienol	Tocotrienols	*Yucca aloifolia*	γ-tocotrienol an inhibits NF-κB pathaway in RAW 264.7 macrophages.	20 µM	[230]	[21]
			γ-tocotrienol treatment in TNF-α-induced inflammation in adipocytes suppressed IκB-α phosphorylation and NF-κB activation.	Cells were pretreated with 0.024, 0.24, and 2.4 μM γ-tocotrienol for 6 h.	[231]	
			In rats with induced arthritis, treatment with γ-tocotrienol reduced arthritis-induced changes in the C-reactive protein, TNF-α, SOD, and GSH levels.	The rats were treated orally with 5 mg/kg body weight of γ-tocotrienol between days 21 and 45.	[232]	
δ-Tocotrienol	Tocotrienols	*Yucca aloifolia*	δ-tocotrienol inhibits NO production and expression of IL-1β, IL-1α, IL-6, TNF-α, IL-12, iNOS, VCAM1 (Vascular cell adhesion protein 1), ICAM1, COX2, IL-1RA, TRAF1 and CD40 in mice.	Mice were fed 100 ppm tocotrienol	[233]	[21]
Succinic acid	Dicarboxylic acid	*Yucca carnerosana*	The injection of succinic acid in Wistar rats showed a high inhibitory capacity of LPO.	25 mg/kg intraperitoneally injected into rats	[234]	[105]
Neophytadiene	Terpenes	*Yucca aloifolia variegata*	Neophytadiene was treated with LPS-stimulated RAW 264.7 and pretreated in rats injected with 10 mg/kg LPS. Neophytadiene inhibited NO production and the expression of TNF-α, IL-6, and IL-10,	25, 50, 100 μM/mL in vitro 12, 25, 50 mg/kg pretreated for 7 days in vivo	[235]	[87]
Mangiferin	Xanthones	*Yucca elephantipes*	Mangiferin pretreatment increases GST, GPx, SOD, and CAT levels in rats with induced myocardial infarction.	100 mg/kg suspended in 2 mL dimethyl sulfoxide administered intraperitoneally for 28 days	[236]	[79]
			Mangiferin pretreatment prevented apoptosis and decreased levels of MDA, IL-1F, and IL-18 in mice subjected to cecal ligation and puncture. This is due to the inhibition of the NLRP3 inflammasome and the upregulation of Nrf2.	15 mg/kg	[237]	
			Mangiferin pretreatment increases HO-1 expression and activity in sepsis-induced mice with acute lung injury. This inhibited MAPK and NF-κB signaling.	10, 30, and 100 mg/kg once daily for 7 days before cecal ligation and puncture.	[238]	
Cinnamaldehyde	Terpenes	*Yucca aloifolia variegata*	Cinnamaldehyde increased antioxidant enzymes such as SOD, GPx, and GST.	Rats were administered orally by gavage at dose levels of 2.14, 6.96, 22.62, and 73.5 mg/kg body weight/day for a period of 10, 30, and 90 days.	[239]	[87]
			Cinnamaldehyde reduced levels of proinflammatory cytokines and increased levels of antioxidant enzymes in patients with rheumatoid arthritis. Molecular docking indicated that cinnamaldehyde interacts with the key residues of TNF-α and IL-6.	20 or 40 µM cinnamaldehyde	[240]	
Nerolidol	Terpenes	*Yucca elata* *Yucca filamentosa*	Nerolidol pretreatment increases SOD, CAT, and GPx, activity by enhancing MP-activated protein kinase phosphorylation.	A significant effect started at 30 μmol/kg	[241]	[59]
			Nerolidol decreased the expression of TNF-α, IL-1β, IL-6, NF-kB, PGE-2, and COX-2 and increased the level of IL-10, IL-4, and serum, antioxidant activity in rats with arthritis induced.	Doses of 200, 400, and 800 m,g/kg were administered for 28 days.	[242]	
Chrysophanol	Anthraquinone	*Yucca elephantipes*	Chrysophanol reduced protein expression of NF-κBp65, p-NF-κB p65, IκBα, p-IκBα, TNF-α, and IL-1β, decreased MPO and MDA levels, and increased SOD activity in mice with acute lung injury (LPS). This by regulating HMGB1/NF-κB signaling through histone deacetylase 3.	Doses of 7.5, 15 and 30 mg/kg	[243]	[79]
			Chrysophanol increased Nrf2 levels and decreased HO-1, glutamate-cysteine ligase, glutamate cysteine ligase, IKKa, IkBa, NF-kB, and ROS generation in H9C2 cells stimulated with LPS. Chrysophanol is suggested to act as an activator of Nrf2.	Cells were cultured at doses of 0, 10, 20, 40, 80, 160, and 320 uM.	[159]	

Table 5 abbreviations: MDA = Malondialdehyde, GPx = Glutathione peroxidase, CAT = Catalase, SOD = Superoxide dismutase, MAPK = Mitogen-Activated Protein Kinase, NF-κB = Nuclear transcription factor-κB, TNF-α = Tumor necrosis factor α, PGE2 = Prostaglandin E2, COX-2 = Cyclooxygenase-2, COX-1 = Cyclooxygenase-1, ROS = Reactive Oxygen, Nrf2 = Nuclear factor erythroid 2–related factor 2, IL = Interleukin, NO = Nitric oxide, ERK = Extracellular signal-regulated kinase, LPO = Lipid Peroxidation, iNOS = Inducible nitric oxide synthase, MPO = Myeloperoxidase, GSH = Glutathione, HO- 1 = Heme oxygenase 1, IKB-α = Inhibitor of nuclear factor kappa B, LPS = Lipopolysaccharide, MIP-2 = Macrophage inflammatory protein-2, IKK = IkB kinase, JNK = Jun N-terminal kinase, ICAM-1 = Intercellular adhesion molecule, GST = Glutathione-S-transferase, FGF2 = Fibroblast growth factor 2, MMP-3 = Matrix Metallopeptidase 3, MMP-13 = Matrix Metallopeptidase 13, ADAMTS- 4 = ADAM Metallopeptidase with Thrombospondin Type 1 Motif 4, TBARS = Thiobarbituric acid reactive substances, ROS = Reactive oxygen species, VCAM1 = Vascular cell adhesion protein 1.

**Table 6:** Quantity of reported phytochemicals in the *Yucca* genus.

Metabolite	*Yucca* Specie	Plant Material	Extraction	Concentration	Reference
Resveratrol	*Yucca schidigera*	Bark was obtained from Desert King Int., Chula Vista, CA, USA	Ultrasonic	9.11 ± 2.11 mg/g plant	[77]
	*Yucca schidigera*	Yucca bark was collected in October 2001 in San Diego, CA.	Soxhlet	2.5 mg/300 g of Yucca powder	[68]
3,3’,5,5’-tetrahydroxy-4-methoxystilbene	*Yucca schidigera*			5.1 mg/300 g of Yucca powder	
	*Yucca schidigera*	Bark was obtained from Desert King Int., Chula Vista, CA, USA	Ultrasonic	9.10 ± 1.10 mg/g plant	[68]
Yuccaol A	*Yucca schidigera*			14.72 ± 2.10 mg/g plant	
	*Yucca gloriosa* roots	The roots and the bark were collected in December, in Tbilisi, Georgia.	MeOH at room temperature	0.02 ± 0.01 mg/g plant	
	*Yucca gloriosa*			2.64 ± 0.80 mg/g plant	
	*Yucca schidigera*	Yucca bark was collected in October 2001 in San Diego, CA.	Soxhlet	3.1 mg/300 g of Yucca powder	[68]
Yuccaol C	*Yucca schidigera*			8.0 mg/300 g of Yucca powder	
	*Yucca gloriosa* roots	The roots and the bark were collected in December, in Tbilisi, Georgia.	MeOH at room temperature	10.68 ± 1.01	[77]
	*Yucca gloriosa*			13.85 ± 1.21 mg/g plant	
	*Yucca schidigera*	Bark was obtained from Desert King Int., Chula Vista, CA, USA	Ultrasonic	12.02 ± 2.28 mg/g plant	
Gloriosaol A	*Yucca gloriosa* roots	The roots and the bark were collected in December, in Tbilisi, Georgia	MeOH at room temperature	3.02 ± 1.50 mg/g plant	[77]
	*Yucca gloriosa*			11.87 ± 1.32 mg/g plant	
Gloriosaol B	*Yucca gloriosa* roots			5.30 ± 1.80 mg/g plant	
	*Yucca gloriosa*			29.73 ± 2.15 mg/g plant	
Gloriosaol C	*Yucca gloriosa* roots			3.33 ± 0.9 mg/g plant	
	*Yucca gloriosa*			4.53 ± 1.42 mg/g plant	
Gloriosaol D & Gloriosaol E	*Yucca gloriosa* roots			4.96 ± 1.71 mg/g plant	
	*Yucca gloriosa*			10.85 ± 1.58 mg/g plant	
Gallic acid	*Yucca elephantipes flowers*	Collected during summer in the city of Xalapa, Veracruz, México	MeOH at room temperature	5.28 μg/g of dry sample	[79]
Protocatechuic acid				21.14 μg/g of dry sample	
Vanillic acid				205.28 μg/g of dry sample	
Chlorogenic acid				47.52 μg/g of dry sample	
Caffeic acid				74.1 μg/g of dry sample	
Ferulic acid				140.59 μg/g of dry sample	
Rutin				393.2 μg/g of dry sample	
Quercetin				9.54 μg/g of dry sample	
Kaempferol				37.16 μg/g of dry sample	

## Data Availability

All data is contained and referenced within the article.

## Abbreviations

TNF-α	Tumor necrosis factor-α
IL-6	Interleukin-6
iNOS	Inducible nitric oxide synthase
IL-1β	Interleukin 1β
GPx	Glutathione peroxidase
CAT	Catalase
SOD	Superoxide dismutase
MAPK	Mitogen-Activated Protein Kinase
NF-κB	Nuclear factor kappa B
Nrf2	Nuclear factor erythroid 2–related factor
FDA	Food and Drug Administration
ROS	Reactive oxygen species
RNS	Reactive nitrogen species
MDA	Malonaldehyde
SIRT1	Sirtuin 1
PGE2	Prostaglandin E2
COX-2	Cyclooxygenase-2
IC50	Half maximal inhibitory concentration
8-oxo-dG	8-Oxo-2’-deoxyguanosine
MMP-9	Matrix metalloproteinase 9
TEAC	Trolox equivalent antioxidant capacity
NLRP3	Nucleotide-binding oligomerization domain-like receptor containing domain 3 of pyrin
PDE4	Phosphodiesterase 4
NO	Nitric oxide
ERK	Extracellular signal-regulated kinase
LPO	Lipid Peroxidation
TLR4	Toll-like receptor 4
MCP-1	Monocyte chemoattractant protein 1
MPO	Myeloperoxidase
GSH	Glutathione
HO-1	Heme oxygenase 1
IκBα	Inhibitor of nuclear factor kappa B
LPS	Lipopolysaccharide
MIP-2	Macrophage inflammatory protein 2
IKK	IkB kinase
JNK	c-Jun amino terminal kinase
ICAM-1	Intercellular adhesion molecule
VEGF	Vascular endothelial growth factor
GST	Glutathione-S-transferase
CRP	Reactive C-protein
PAR-1	Protease-activated receptor 1
TXB2	Thromboxane B2
IgE	Immunoglobulin E
TBARS	Thiobarbituric acid-reactive substances
PKA	Protein kinase A
mTOR	Mammalian target of rapamycin
EGFR	Epidermal growth factor receptor
Keap1	Kelch-like ECH-associated protein 1
COX-1	Cyclooxygenase-1
VCAM1	Vascular cell adhesion protein 1
VWF	von Willebrand factor
GPIbα	platelet adhesion receptor glycoprotein
AA	arachidonic acid
TxA2	Thromboxane A2
IL-13	Interleukin-13
NOS	Nitric oxide synthase
PUFAs	Polyunsaturated fatty acids
PEs	Phosphatidylethanolamines
IL-8	Interleukin 8
IL-1RI	IL-1 type I receptor
IL-1RAcP	IL-1R accessory protein
H_2_O_2_	Hydrogen peroxide
NIK	NF-kB inducing kinase
TRAF	TNF receptor-associated factor
RTK	Receptor tyrosine kinase
Cul3	Cullin 3
NQO1	NADP(H) quinone oxidoreductase
ORAC	Oxygen radical absorbance capacity
DPPH	1,1-diphenyl-2-picrylhydrazyl
